# Intraoperative CT-guided navigation versus fluoroscopy for percutaneous pedicle screw placement in 192 patients: a comparative analysis

**DOI:** 10.1186/s10195-022-00661-8

**Published:** 2022-09-01

**Authors:** Giuseppe La Rocca, Edoardo Mazzucchi, Fabrizio Pignotti, Luigi Aurelio Nasto, Gianluca Galieri, Alessandro Olivi, Vincenzo De Santis, Pierluigi Rinaldi, Enrico Pola, Giovanni Sabatino

**Affiliations:** 1 Department of Neurosurgery, Mater Olbia Hospital, Olbia, Italy; 2 Institute of Neurosurgery, IRCCS Fondazione Policlinico Universitario Agostino Gemelli, Catholic University, Rome, Italy; 3 Neurosurgical Training Center and Brain Research, Mater Olbia Hospital, Olbia, Italy; 4 Institute of Orthopedics, IRCCS Fondazione Policlinico Universitario Agostino Gemelli, Catholic University of Rome, Rome, Italy; 5 Unit of Orthopedics, Mater Olbia Hospital, Olbia, Italy; 6 Unit of Radiology, Mater Olbia Hospital, Olbia, Italy; 7 Unit of Orthopedics and Spine Surgery, Primo Policlinico Di Napoli, Università Della Campania Luigi Vanvitelli, Piazza Luigi Miraglia, 2, 80138 Naples, Italy

**Keywords:** Minimally invasive spine surgery, Percutaneous pedicle screws, CT navigation, Degenerative spondylolisthesis, Low-back-pain surgery, Radiation exposure

## Abstract

**Background:**

Percutaneous pedicle screw (PPS) placement is a key step in several minimally invasive spinal surgery (MISS) procedures. Traditional technique for PPS makes use of C-arm fluoroscopy assistance (FA). More recently, newer intraoperative imaging techniques have been developed for PPS, including CT-guided navigation (CTNav). The aim of this study was to compare FA and CTNav techniques for PPS with regard to accuracy, complications, and radiation dosage.

**Materials and methods:**

A total of 192 patients with degenerative lumbar spondylolisthesis and canal stenosis who underwent MISS posterior fusion ± interbody fusion through transforaminal approach (TLIF) were retrospectively reviewed. Pedicle screws were placed percutaneously using either standard C-arm fluoroscopy guidance (FA group) or CT navigation (CTNav group). Intraoperative effective dose (ED, mSv) was measured. Screw placement accuracy was assessed postoperatively on a CT scan using Gertzbein and Robbins classification (grades A–E). Oswestry disability index (ODI) and visual analog scale (VAS) scores were compared in both groups before and after surgery.

**Results:**

A total of 101 and 91 procedures were performed with FA (FA group) and CTNav approach (CTNav group), respectively. Median age was 61 years in both groups, and the most commonly treated level was L4–L5. Median ED received from patients was 1.504 mSv (0.494–4.406) in FA technique and 21.130 mSv (10.840–30.390) in CTNav approach (*p* < 0.001). Percentage of grade A and B screws was significantly higher for the CTNav group (96.4% versus 92%, *p* < 0.001), whereas there were 16 grade E screws in the FA group and 0 grade E screws in the CTNav group (*p* < 0.001). A total of seven and five complications were reported in the FA and CTNav group, respectively (*p* = 0.771).

**Conclusions:**

CTNav technique increases accuracy of pedicle screw placement compared with FA technique without affecting operative time. Nevertheless, no significant difference was noted in terms of reoperation rate due to screw malpositioning between CTNav and FA techniques. Radiation exposure of patients was significantly higher with CTNav technique.

**Level of Evidence:** Level 3.

## Introduction

Minimally invasive spine surgery (MISS) and percutaneous pedicle screw (PPS) placement in degenerative lumbar spondylolisthesis with lumbar canal stenosis (DLSS) is a routinely used and widely accepted technique. Although several MISS techniques are available, the shared goals of these procedures are to offer shorter operative time and hospitalization, minimize blood loss, reduce muscular damage and low back pain, and decrease rate of postoperative complications compared with open surgery [1–5]. Pedicle screw placement is a key step in all MISS procedures. Accurate placement of pedicle screws is of the utmost importance to achieve stable fixation of the spine and avoid any neurological damage. Over the years, new image-guided techniques have been developed to overcome some of the limitations of the traditional fluoroscopy assisted (FA) technique for pedicle screw placement. To date, no definitive study has been performed to compare FA technique with newer systems such as CT-guided technique. The aim of the current study was to compare screw accuracy placement and radiation exposure of fluoroscopy-assisted (FA) versus CT navigation-guided (CTNav) technique for PPS placement in DLSS.

## Materials and methods

### Patients selection and demographics

Following approval by our local ethical committee (no. 276/2020/CE), we conducted a retrospective review of 192 consecutive patients who underwent lumbar arthrodesis with PPS for DLSS with two different techniques: fluoroscopy-assisted (FA group) and CT navigation (CTNav group). Surgical procedures were performed by the senior author (G.S.) and his assistant (G.L.R.). All patients signed a written informed consent before surgery. Inclusion criteria for the study were: (1) age at surgery between 18 and 75 years, (2) low back pain (LBP) with radicular irradiation in the lower limbs, (3) claudicatio neurogena, and (4) failed conservative treatment for at least 6 months. Diagnosis of DLSS was confirmed with standard standing AP and lateral X-ray of the lumbar spine, flexion–extension X-rays, and lumbar spine magnetic resonance imaging (MRI). Patients with a previous history of instrumented spine surgery as well as osteopenia defined as lumbar T-score < −1 SD on dual energy X-ray absorptiometry were excluded from the study. All patients operated on before March 2020 were assigned to the FA group; after March 2020, all patients were operated on with CT navigation technique. Demographics, intraoperative, clinical outcome, and radiological data were recorded.

### Surgical technique

Surgery was performed under general anesthesia with patients positioned prone on a radiolucent table (TruSystem 7000, TRUMPF Medizin Systeme GmbH, Saalfeld, Germany). Intraoperative neuromonitoring was used for all surgical procedures (NVM5, NuVasive, Memphis, TN, USA). In the FA group, a standard C-arm fluoroscopy (OEC Brivo Plus, GE Healthcare, USA) was used for pedicle screw placement. We proceeded from rostral to caudal, putting both screws of the same level at the same time to reduce intraoperative radiation exposure. In the CTNav group, an AIRO mobile intraoperative CT scanner (v 2.1.0.2, Mobius Imaging LLC, Shirley, MA, USA) was used for pedicle screw placement. In brief, a small midline lumbar incision at the level of the intercristal line [6] was performed, and a spinous process clamp (Brainlab AG, Munich, Germany) was placed. The spinous process clamp was used as a reference guide for CT scanning. Following 3D reconstruction of the surgical area, a navigated drill guide (Brainlab AG, Munich, Germany) was used to drill the holes for PPS placement. After PPS placement, an intraoperative CT scan was performed again to check for the accuracy of screws placement. After screw placement, a midline incision for laminoartrectomy and dural sac/roots decompression was performed in both groups. Transforaminal interbody fusion with TLIF cage (Trabecular Metal, TM Ardis, Zimmer Biomet, IN, USA) was added where deemed appropriate. At the end of the surgical procedure, AP and lateral X-ray was performed to confirm the correct position of the implants in all patients.

### Radiological and clinical outcomes

Intraoperative radiation exposure data were collected according to the imaging modality used. In the FA group, radiation exposure was recorded by the C-arm software in terms of: (1) cumulative radiation exposure (mGy), defined as the kinetic energy per unit mass of air provided to a defined point in space, (2) dose–area product (DAP) (Gy·cm^2^), defined as patient’s dose per area of skin irradiated within the radiation field, and (3) radiation time (seconds) total time of X-ray beam activation [7]. In the CTNav group, radiation exposure was measured using BrainLab Curve 1.2 navigation system software (Brainlab AG, Munich, Germany) provided with the AIRO Mobile intraoperative CT scanner (Mobius Imaging LLC, Shirley, MA, USA). Radiological data from AIRO included (1) dose–length product (DLP) (mGy·cm), a measure of CT tube radiation output, and (2) CT dose index volume (CTDIvol) (mGy), the radiation intensity used to perform a specific CT exam. To compare radiation exposure between the two groups, effective dose (ED) in mSv was calculated. In FA group, ED was calculated by multiplying DAP measurement by the conversion factor 0.27 mSv/Gy·cm^2^, as previously reported [8, 9]. Similarly, in the CTNav group, ED was calculated by multiplying DLP by the dose–length conversion factor 0.014, as previously reported [10–12].

All patients underwent X-ray and CT scan examination at 6 months after surgery. Screw positioning was assessed by a radiologist and a spine surgeon not involved in the surgical care of the patients. Screw placement accuracy was measured according to Gertzbein and Robbins classification and was graded from A to E, according to the extent by which every single screw breached the cortex of the pedicle [13, 14]. Screws were graded as follows: (A) fully intrapedicular position without breach of the pedicle cortex; (B) breach of the pedicle cortex < 2 mm; (C) breach of the pedicle cortex 2–4 mm; (D) breach of the pedicle cortex 4–6 mm; (E) breach of the pedicle cortex > 6 mm or screw outside of the pedicle. Grades A and B were considered satisfactory results, whereas grades C–E were considered unsatisfactory results.

All patients were asked to complete Oswestry disability index (ODI), visual analog scale leg pain (VAS-LP), and visual analog scale back pain (VAS-BP) questionnaires before surgery and at 6-month follow-up.

### Statistical analysis

Statistical analysis was performed using GraphPad Prism 5.01 software (GraphPad Software Inc., San Diego, CA). Radiological and clinical data were expressed as median (range) and count (percentages), as appropriate. Means and percentages between the two groups were compared using the Mann–Whitney *U* and the *χ*^2^ tests, as appropriate. A *p* value < 0.05 was considered significant.

## Results

### Demographics and operative data

One-hundred and ninety-two MISS procedures with PPS placement for degenerative lumbar spondylolisthesis with stenosis (DLSS) were performed between August 2019 and March 2021 at our institution. From August 2019 to February 2020, 101 procedures were performed with FA technique (FA group), while from March 2020 to March 2021, 91 cases were performed with CTNav approach (CTNav group). Patients demographics are summarized in Table 1. Median age was 61 years in both groups (*p* = 0.811), and male-to-female ratio was also similar (*p* = 0.664). There were no statistically significant differences between the two study groups in terms of body mass index (BMI) or smoking habit. Fourteen (13.9%) and 21 (23.1%) patients had a history of previous uninstrumented spinal surgery in FA and CTNav group, respectively (*p* = 0.098). Minimum follow-up was 6 months, but length of follow-up after surgery was longer for the FA group because patients in this group were operated on earlier.

**Table 1:** Comparison of demographic and clinical data between patients operated with FA (*n* = 101) and CTNav (*n* = 91) technique

	FA group (*n* = 101)	CTNav group (*n* = 91)	*p*
Sex (M:F)	56:45	47:44	0.664
Age (years)	61 (23–75)	61 (37–75)	0.811
BMI (kg/m^2^)	27.0 (17.6–36.4)	26.9 (17.7–42.1)	0.745
Smoking (yes:no)	45:56	33:58	0.303
Previous spinal surgery (%)	14 (13.9)	21 (23.1)	0.134
Length of hospital stay (days)	2 (2–6)	2 (2–5)	0.184
Length of follow-up (days)	483 (321–791)	253 (182–588)	** < 0.010**

Data are reported as median (range), counts are used where appropriate. *FA* fluoroscopy assisted, *CTNav* CT-navigation assisted, *M* male, *F* female, *BMI* body mass index

### Surgical data

The most commonly treated level was L4–L5, followed by L3–L5 in both groups. There were no statistically significant differences in terms of distribution and number of treated segments between the two study groups. (Table 2).

**Table 2:** Comparison of the operated levels between FA (*n* = 101) and CTNav (*n* = 91) groups

	FA group (*n* = 101)	CTNav group (*n* = 91)	*p*
Single-level surgery (%)	59 (58.4)	53 (58.2)	0.980
L2–L3 (%)	0 (0.0)	1 (1.1)	
L3–L4 (%)	4 (4.0)	6 (6.6)	
L4–L5 (%)	38 (37.6)	38 (41.7)	
L5–S1 (%)	17 (16.8)	8 (8.8)	
Two-level surgery (%)	35 (34.7)	33 (36.3)	0.815
L2–L4 (%)	3 (3.0)	3 (3.3)	
L3–L5 (%)	20 (19.8)	17 (18.7)	
L4-S1 (%)	12 (11.9)	13 (14.3)	
Three-level surgery (%)	7 (6.9)	5 (5.5)	0.681
L2–L5 (%)	5 (4.9)	4 (4.4)	
L3–S1 (%)	2 (2.0)	1 (1.1)	

Data are reported as counts (percentages). *FA* fluoroscopy assisted, *CTNav* CT-navigation assisted

A total of 952 PPSs were placed during the study period, 502 screws with FA technique and 450 screws with CTNav. Median screw number per patient was 4 (4–8) for both techniques (*p* = 0.83). A TLIF fusion was added in 40 (39.6%) and 50 (54.9%) patients in FA and CTNav groups, respectively (*p* = 0.043). No significant difference was observed in terms of total duration of surgery and time per single screw placement between the two groups. The only significant difference was observed for time per screw placement in single-level surgery (6 min in FA group versus 7 min in CTNav group, *p* = 0.032). A total of seven complications were reported in the FA group (three unintended dural tears during decompression, three cases of postoperative anemia that required transfusion, one TLIF cage mobilization that required revision surgery). In the CTNav group, a total of five complications were reported (two unintended dural tears during decompression, and three cases of postoperative anemia that required transfusion) (*p* = 0.771). (Table 3).

**Table 3:** Surgical data comparison between FA group (*n* = 101) and CTNav group (*n* = 91)

	FA group (*n* = 101)	CTNav group (*n* = 91)	*p*
Total number of screws	502	450	–
Screws per patient	4 (4–8)	4 (4—8)	0.830
TLIF (yes:no)	40:61	50:41	**0.043**
Operative time (min)	145 (55–320)	155 (90–290)	0.060
Time per single screw (min)	5.7 (2.5–17.7)	6.3 (3.1–14.7)	0.154
Time per screw—1 level (min)	6.0 (2.5–17.7)	7.0 (4.0–14.7)	**0.032**
Time per screw—2 levels (min)	5.5 (3.2–10.2)	5.3 (4.0–7.7)	0.973
Time per screw—3 levels (min)	5.6 (3.0–7.6)	3.9 (3.1–6.4)	0.224
Complications (%)	7 (6.9)	5 (5.5)	0.771

Statistically significant differences between the two study groups (*p* < 0.05) are highlighted in bold

Data are expressed as median (range) unless stated otherwise. *FA* fluoroscopy assisted, *CTNav* CT-navigation assisted, *TLIF* transforaminal lumbar interbody fusion

### Radiological and clinical outcomes

Median ED received from patients was 1.504 (0.494–4.406) mSv in the FA technique and 21.130 (10.840–30.390) mSv in the CTNav approach (*p* < 0.001). As per Gertzbein and Robbins classification [13], the percentage of grade A and B screws was significantly higher for the CTNav group (96.4% versus 92%, *p* < 0.001). Numbers of grade C and D screws were not significantly different between the two groups, whereas there were 16 grade E screws in the FA group and 0 grade E screws in the CTNav group (*p* < 0.001) (Table 4).

**Table 4:** Radiation dose exposure and screw placement accuracy comparison

	FA group (*n* = 101)	CTNav group (*n* = 91)	*p*
ED (mSv)	1.504 (0.494–4.406)	21.130 (10.840–30.390)	< 0.001
Screw placement Gertzbein–Robbins classification	502	450	< 0.001
A	390 (77.7)	417 (92.7)	< 0.05*
B	72 (14.3)	17 (3.7)	< 0.05*
C	18 (3.6)	12 (2.7)	ns
D	6 (1.2)	4 (0.9)	ns
E	16 (3.2)	0 (0.0)	< 0.05*

Data are expressed as median (range) and count (percentage) as appropriate.

*FA*, fluoroscopy assisted, *CTNav* CT-navigation assisted, *ED* effective dose, *mSv* milliSievert.

*p values from *z*-test comparison with Bonferroni correction

Patient reported outcomes measures in terms of ODI, VAS leg pain, and VAS back pain are presented in Table 5. In both groups, a significant improvement in scores was observed after surgery.

**Table 5:** Patient-reported outcome measures before and after surgery (6 months follow-up)

	Before surgery	After surgery	*p*
VAS leg pain			
FA group	9 (4–10)	2 (0–10)	< 0.010
CTNav group	8 (4–10)	2 (0–7)	< 0.010
VAS back pain			
FA group	9 (5–10)	3 (0–10)	< 0.010
CTNav group	8 (4–10)	4 (0–9)	< 0.010
ODI			
FA group	57 (16–92)	27 (0–68)	< 0.010
CTNav group	53 (14–82)	34 (6–80)	< 0.010

Data are expressed as median (range). *VAS* visual analog scale, *ODI* Oswestry disability index, *FA* fluoroscopy assisted, *CTNav* CT-navigation assisted

## Discussion

In recent years, tremendous advancements have been made in the development of new spine stabilization systems together with new image-guidance techniques. Accuracy in pedicle screw placement is of the utmost importance to achieve a stable fixation and avoid neurological damage. Several surgical techniques have been described for pedicle screw placement, the most common ones being: (1) freehand, (2) fluoroscopy-assisted, (3) CT navigation-guided, and (4) robot-assisted [15–24]. Although no definitive comparative study has been performed on these techniques, more recent techniques are generally perceived as being safer and more accurate. On the other hand, one potential drawback of more recent techniques is that they rely heavily on the use of intraoperative radiation. This has raised some concerns with regard to radiation exposure for patients and surgical teams [25–27].

In a recent meta-analysis by Perdomo-Pantoja et al., the use of CTNav guidance for PPS placement showed the highest accuracy (95.5%) compared with freehand (93.1%), fluoroscopy-assisted (91.5%), and robot-assisted (90.5%) techniques. According to the authors, robot-assisted CTNav showed the highest accuracy rate in thoracic spine compared with freehand technique [23]. In the present study, we compared two of the most commonly used techniques for PPS placement in DLSS: traditional C-arm fluoroscopy versus intraoperative CT guided. Our data show that the two techniques are similar in terms of operative time and time per screw in two- and three-level operations, while in one-level surgery screw placement time was lower for FA technique. This can be explained by the fact that the C-arm does not need any setup for reuse during the same procedure, whereas CT-guided systems need some extra setup time between scans (e.g., pre- and post-screw placement) for multiple-level surgery. Accuracy of screw placement was significantly higher for the CTNav group (96.4% A and B) than the FA group (92% A and B) [23, 29]. Furthermore, 16 grade E screws were detected in FA group, although none of them resulted in any new-onset neurological deficit or required revision surgery. Interestingly, in CTNav group, six patients underwent a third intraoperative CT scan because six screws were classified as grade E on the first intraoperative scan and required replacement (after screw replacement, three screws were graded as B and three as C).

With regard to radiation exposure, our findings are in line with similar reports available in literature for both FA and CTNav technique [24]. ED was significantly higher for CTNav technique, although the surgical team was not exposed to any additional radiation. FA technique exposes the patient to a lower radiation dose, but the surgical team cannot leave the operating room during fluoroscopy operation and thus is exposed to the same amount of radiation of the patients. One additional factor to consider when comparing these two techniques is the expertise of the surgeon: experienced surgeons tend to use lower fluoroscopy, and this can significantly impact the total effective dose. Intraoperative CT is fixed, and radiation dose depends on patient features only.

Our study has some limitations. The first limitation is its retrospective nature. FA procedures were performed before CTNav procedures, and some learning curve effect should be taken into account when analyzing our data. Finally, although large enough for the current study, our sample size is insufficient to draw general conclusions with regard to longer fusions (i.e., > three levels). Although it is not possible, on the basis of our data, to strictly recommend one technique over the other, we believe that, if CT navigation technology, is available it makes total sense to use it for every routine case. Furthermore, the technique can be particularly useful in patients with obesity, patients with dysplastic pedicles, or in revision cases where normal anatomy has been disrupted by previous surgery.

In conclusion, CTNav technique is a safe adjunct to spinal surgery. It reduces surgeon and staff radiation exposure (although it does increase radiation dose for patients) and increases the accuracy of screw placement without affecting operation time. Nevertheless, no significant difference was noted in terms of reoperation rate due to screw malpositioning between CTNav and FA techniques.

## Data Availability

The datasets generated during the current study are available from the corresponding authors upon request.

## Abbreviations

MISS: Minimally invasive spine surgery
PPS: Percutaneous pedicle screw
DLSS: Degenerative lumbar spondylolisthesis and lumbar canal stenosis
FA: Fluoroscopy assisted
CTNav: CT-navigation assisted
LBP: Low back pain
MRI: Magnetic resonance imaging
TLIF: Transforaminal lumbar interbody fusion
DAP: Dose–area product
DLP: Dose–length product
CTDIvol: CT dose index volume
ED: Effective dose
ODI: Oswestry disability index
VAS-LP: Visual analog scale leg pain
VAS-BP: Visual analog scale back pain
BMI: Body mass index
